# Metformin attenuates LPS-induced neuronal injury and cognitive impairments by blocking NF-κB pathway

**DOI:** 10.1186/s12868-021-00678-5

**Published:** 2021-11-26

**Authors:** Chenliang Zhou, Bo Peng, Zhenghui Qin, Wei Zhu, Cuiping Guo

**Affiliations:** 1 Department of Critical Care Medicine, Renmin Hospital of Wuhan University, Wuhan, China; 2 Department of Neurology, Renmin Hospital of Wuhan University, Wuhan, China; 3 Department of Critical Care Medicine, Tianyou Hospital Affiliated to Wuhan University of Science and Technology, Wuhan, China

**Keywords:** Lipopolysaccharides (LPS), Metformin, NF-κB, Neuronal injury, Cognitive impairments

## Abstract

**Background:**

Neuroinflammatory response is considered to be a high-risk factor for cognitive impairments in the brain. Lipopolysaccharides (LPS) is an endotoxin that induces acute inflammatory responses in injected bodies. However, the molecular mechanisms underlying LPS-associated cognitive impairments still remain unclear.

**Methods:**

Here, primary hippocampal neurons were treated with LPS, and western blotting and immunofluorescence were used to investigate whether LPS induces neurons damage. At the same time, SD rats were injected with LPS (830 μg/Kg) intraperitoneally, and Open field test, Novel Objective Recognition test, Fear condition test were used to detect cognitive function. LTP was used to assess synaptic plasticity, and molecular biology technology was used to assess the NF-κB pathway, while ELISA was used to detect inflammatory factors. In addition, metformin was used to treat primary hippocampal neurons, and intraventricularly administered to SD rats. The same molecular technics, behavioral and electrophysiological tests were used to examine whether metformin could alleviate the LPS-associated neuronal damage, as well as synaptic plasticity, and behavioral alterations in SD rats.

**Results:**

Altogether, neuronal damage were observed in primary hippocampal neurons after LPS intervention, which were alleviated by metformin treatment. At the same time, LPS injection in rat triggers cognitive impairment through activation of NF-κB signaling pathway, and metformin administration alleviates the LPS-induced memory dysfunction and improves synaptic plasticity.

**Conclusion:**

These findings highlight a novel pathogenic mechanism of LPS-related cognitive impairments through activation of NF-κB signaling pathway, and accumulation of inflammatory mediators, which induces neuronal pathologic changes and cognitive impairments. However, metformin attenuates LPS-induced neuronal injury and cognitive impairments by blocking NF-κB pathway.

**Supplementary Information:**

The online version contains supplementary material available at 10.1186/s12868-021-00678-5.

## Introduction

Neuroinflammation is a double-edged sword. On one hand, inflammatory mechanisms are beneficial in the repair of nervous system injury in certain circumstances. However, on the other hand, prolonged neuroinflammation could induce or at least exacerbate neurodegenerative diseases [1–3]. Clinical studies and animal experiments have shown that neuroinflammation in the brain is closely related to the occurrence and development of various acute and chronic neurodegenerative diseases such as Parkinson’s disease (PD) [4, 5], Alzheimer’s disease (AD) [6, 7] and multiple sclerosis (MS) [8, 9]. However, the role of neuroinflammation in the progression of neurodegenerative diseases is very complex and many problems are far from being clear.

Metformin, a biguanide class of antidiabetics, has shown promise in the management of cognitive impairments [10, 11], as its administration was found to improve mild cognitive impairments in a clinical study [12]. It is well known that cognitive function could be impaired by variousassaults, including inflammation. Neuroinflammation plays an important role in the pathogenesis of cognitive dysfunction after lipopolysaccharides (LPS) exposure [13, 14]. Metformin was reported to improveschizophrenia-like cognitive deficits induced by LPS exposure and reduces LPS-induced fear memory reconsolidation impairments and ameliorated inflammation [15].

NF-κB is an important transcription factor that regulates chronic disease by promoting inflammation [16, 17]. Activated NF-κB was detected in the substantia nigra of PD patients and PD animal models. More importantly, the substantia nigra of PD patients at autopsy showed significant co-localization of NF-κB with p65 and CD11b (markers of microglia cells), suggesting that the inflammatory response regulated by NF-κB plays an important role in the pathogenesis of PD [18, 19]. Moreover, inhibition of NF-κB activation prevented the activation of microglia and the loss of dopaminergic neurons in PD animals [20]. Therefore, these together indicate that NF-κB may be an important therapeutic target in the treatment of neuroinflammatory and neurodegenerative diseases. We therefore, hypothesized that metformin might alleviate LPS-induced cognitive impairments, and NF-κB might play a key role.

In the current study, behavioral tests showed that blocking or decreasing NF-κB activity attenuates LPS-induced learning and memory dysfunction in rats. Moreover, metformin decreases inflammatory response in the hippocampus, restores the reduced expression of synaptic proteins and improves synaptic damage. These data suggest that treatment with metformin may contribute to the recovery of learning and memory function after LPS-induced neuronal injury and cognitive impairments, and provide an insight into clinical intervention in neuropathological and behavioral complications associated with neuroinflammatory disorders.

## Material and methods

### Reagents

LPS was purchased from Sigma Aldrich (*Escherichia coli* serotype 0127:B8, Sigma Aldrich L3129). Nuclear and cytoplasmic protein preparation kit (P1200) was purchased from Pulilai, and all cell culture reagents were from Thermo Fisher Scientific. Rat IL-1β (Interleukin 1 Beta) ELISA Kit (E-EL-R0012c), Rat IL-6(Interleukin 6) ELISA Kit (E-EL-M0044c), Rat TNF-α (Tumor Necrosis Factor Alpha) ELISA Kit (E-EL-R2856c) were purchased from Elabscience Biotechnology.

### Animals

**Fig. 1:**
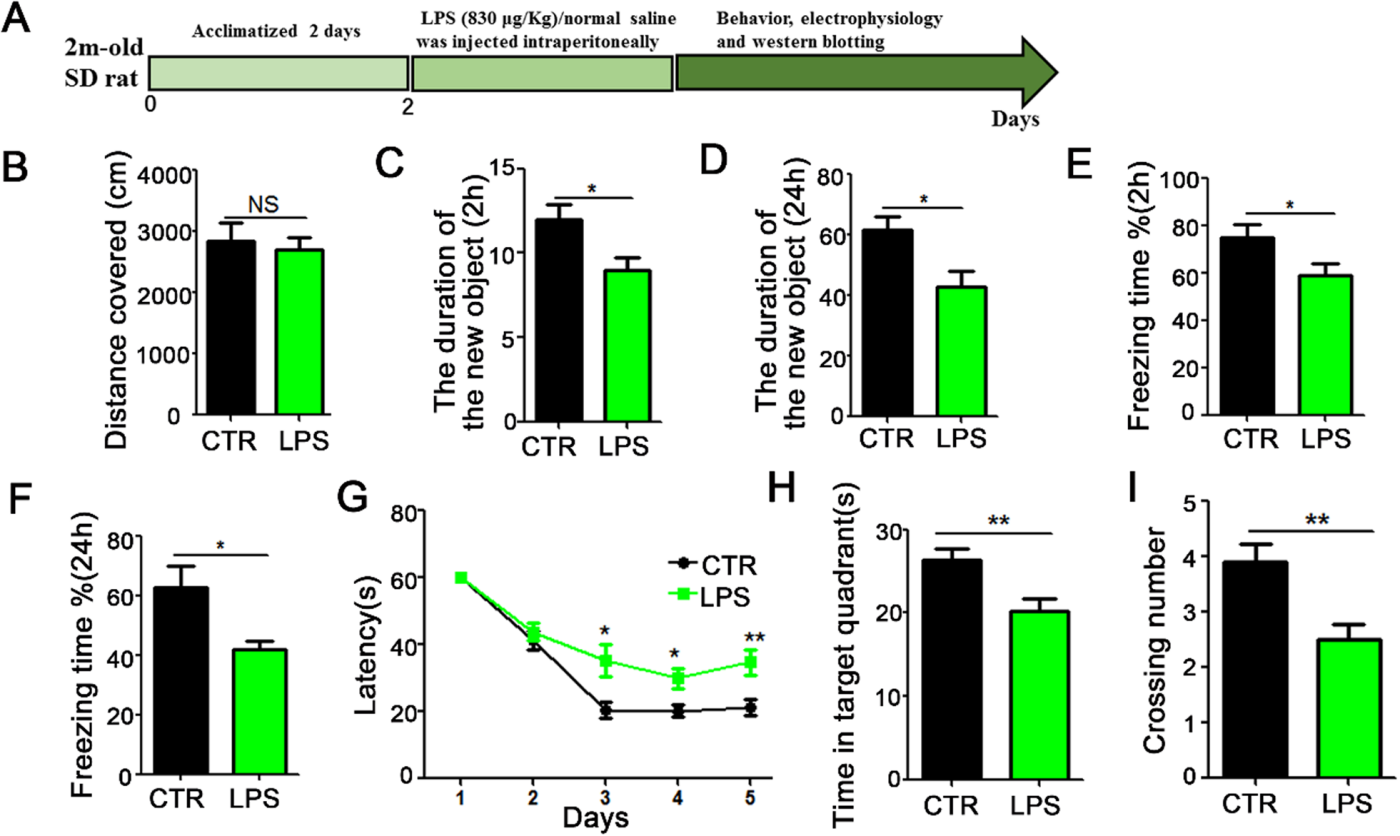
Lipopolysaccharide (LPS) treatment triggers cognitive deficits in rats. **A** Experimental design sketch. **B** The open field showed no difference in the total distance covered. **C**, **D** Novel object recognition test (NOR) showed the measured recognition index of the new object in 2 h (**C**) and 24 h (**D**). **E**, **F** Fear condition test showed the freezing time for 2 h (**E**) and 24 h (**F**). Morris water maze (MWM) showed the latency to find the hidden platform (**G**). Spatial memory was tested with the platform removed.The time in the target quadrant (**H**) and the number of target platform crossing (**I**) were recorded. n = 10. *p* value significance is calculated from a *T*-test, and data are presented as mean ± SEM. *p < 0.05, **p < 0.01 vs control group

Male Sprague–Dawley (SD) rats (2 months old) were supplied by Liaoning Changsheng Biotech Co. Ltd. Rats were fed with normal rats’ food and kept under 12-h light and 12-h dark cycle with free water access. Rats were randomly assigned to different experimental groups and treated according to the experimental requirements. LPS (830 μg/Kg) was injected intraperitoneally (i.p.) and an equal volume of vehicle (normal saline) was used as a control. Behavioral, electrophysiological, and biochemical tests were performed at 6 h after injection (Fig. 1A). Metformin has might have a certain therapeutic potential for cognitive impairments. To further investigate on this, Healthy SD rats were randomly divided into three groups, the control group (control group), model group (LPS group) and intervention group (Met + LPS group). The Met + LPS group were injected with metformin 10 mM into lateral ventricle and LPS (830 μg/Kg) injection intraperitoneally. Behavioral, electrophysiological, and biochemical tests were performed at 6 h after injection (Fig. 5A).

### LPS model

LPS (830 μg/Kg) was injected intraperitoneally (i.p.) and an equal volume of vehicle (normal saline) was used as a control. Behavioral and electrophysiological tests were performed at 6 h after injection. After completion of the tests, the animals were sacrificed, the hippocampi were quickly removed, immediately frozen on dry ice and stored at − 80 °C until further molecular analysis [21].

### Lateral ventricle stereotactic surgery

Rats were anesthetized with isoflurane and placed on a stereotactic device. Bilateral holes were drilled in the skull stereotaxically at the following coordinates: posterior 1.2 mm, lateral 2.6 mm, and depth 4.0 mm relative to bregma. Concentration of 5 mM and 10 mM metformin (5 µL) were injected into the lateral ventricles at a rate of 0.25 μL/min. Vital signs of the rats were monitored. After the injection, the needle was allowed to stay for two minutes and then slowly pulled out.

### Behavior tests

#### Open field test

The rats were put into the laboratory and stayed for two days, and the rats were placed alone in the test box (100 cm × 100 cm × 70 cm container) for spontaneous activity for 5 min. The distance traveled was tracked and measured to evaluate their locomotor ability.

#### Novel objective recognition test

In the same way as open field test, the rats were placed in the laboratory and allowed to stay for one day, before the experimental test was carried out. Object A and B were placed on the two corners of the box, rats are placed in center of the box and allowed to freely explore for 5 min. After 2 h, object B was replaced by the new object C, the rats are placed in the box for another 5 min. After 24 h, object C was replaced by object D, and the rats were again placed in the box for 5 min. The exploration time of objects A, B, C and D were recorded during the activity.

#### Fear conditioning test

Similarly, the rats were put into the testing room one day earlier for adaptation. The experiment was divided into two stages. In the first stage (training), rats were placed in a test box for 3 min, followed by 10 s of sound stimulation, and immediately followed by electrical shock stimulation (0.8 mA, 3 s), repeatedly three times. In the second stage (test), following stimulation completion, the test is performed at 2 h and 24 h. During this process, only the sound stimulus was given, without electrical shock stimulation. The freezing time of rats was recorded during the tests at 2 h and 24 h [22].

#### Morris water maze test (MWM)

Spatial learning and memory were tested by MWM test. The rats were put in the room a day earlier. When testing spatial learning ability, rats were placed in a water maze to find a hidden platform in 60 s for five consecutive days. If the rat did not find the platform within 60 s, they were guided to the platform and allowed to stay there for 20 s. On the 6th day, the platform was removed and spatial memory was tested, the number of crossing the platform position and the time spent in the target quadrant were recorded.

### Long-term potentiation (LTP)

LTP was used to detect synaptic plasticity in the hippocampus, and the MED64 multi-electrode array system was used (Alpha Med Sciences, Kadoma, Japan) to detect the LTP. Before detection, the rats were quickly sacrificed and brains were quickly removed and cut. The slices were incubated in artificial cerebrospinal fluid for 30 min, followed by three sets of high-frequency stimulation (HFS of 100 Hz, lasting for 1 s). The field excitatory postsynaptic potentials (FEPSPs) of CA1 neurons were recorded [22, 23].

### Primary cultures of hippocampal neurons

Primary cultures of hippocampal neurons were performed as previously described [24]. Briefly, hippocampal tissue was quickly removed and placed in HANK’s buffered saline and gently chopped, then suspended in a 0.25% (v/v) trypsin solution at 37 °C for 15 min. Neurons were plated in 6-well and 12-well plates coated with 100 μg/mL poly-D-lysine and supplemented with 2% (v/v) B-27 and 1 × GlutaMAX. Hippocampal neurons were cultured and treated at 37 °C in a humidified 5% (vol/vol) CO_2_ incubator [22].

### Immunofluorescence

Hippocampal neurons were fixed with 4% paraformaldehyde for 8 min, washed three times with PBS, blocked with 3% BSA and 0.5% Triton X-100 for 30 min, and labeled overnight with primary antibody at 4 °C. The second day, the cells were rinsed in PBS for three times. The second antibody was then incubated at room temperature for 1 h, and then rinsed in PBS for three times. The nuclei were stained with Hocest (1:1000) for 10 min. Then the neurons were washed three times with PBS and mounted on slides. Confocal microscopy was used to detect cell morphology (LSM710, Zeiss, Germany) [22].

### Western blotting

Western blotting was used to detect protein expression levels. Cell or tissue samples were lysed with RIPA containing the protease inhibitor PMSF and cocktail, and then centrifuged at 12,000*g* for 10 min. Supernatants were boiled in SDS loading buffer and protein separated using SDS–PAGE. Proteins were then transferred to nitrocellulose membranes. Five percent skim milk powder was used to block, and proteins of interest were labeled overnight with primary antibody at 4 °C. After three times wash with washing buffer, the second antibody was incubated at room temperature for 1 h followed by another three washes, protein expression levels were visualized by using the Odyssey system. The primary antibodies used for Western blotting include PSD95 (1:1000; Cell Signaling Technology, USA), NR1 (1:1000; Millipore, USA), NR2B (1:1000; Millipore, USA), NR2A (1:1000; abcam, UK), GluA1 (1:1000; Millipore, USA), GluA2 (1:1000; Millipore, USA), Synapsin 1(1:1000; Millipore, USA), Synaptophysin (1:1000, sigma), actin (1:1000; Abcam), LaminB1(1:1000; Abcam), NF-κB p65(1:500; Cell Signaling Technology, USA).

The nuclear and cytoplasmic proteins preparation kit (P1200, Pulilai) was used to separate the nuclear and cytoplasmic components according to the manufacturer’s procedures for subsequent experiments.

### Statistical analysis

Data were expressed as mean ± SEM and analyzed using GraphPad statistical software. The one-way ANOVA was used to determine the differences among groups. For the comparison between two groups, the Student’s *t* test was used. The significance was assessed at *p* < 0.05.

## Results

### Lipopolysaccharide (LPS) injection triggers cognitive deficits in rats

LPS is a unique component in the cell wall of gram-negative bacteria [25–27]. LPS can induce a cascade reaction of immune stimulus and toxic pathophysiological activity of the body, releasing endotoxin and causing inflammation [28, 29]. Also, adult male rats injected with LPS showed inflammation in the central nervous system which might affect it normal function. To explore the effect of LPS on cognitive function, 20 healthy SD rats were randomly divided into LPS model group and control group which were respectively intraperitoneally injected with LPS (830 μg/Kg/i.p, n = 10) or normal saline (1 mL/Kg/ip, n = 10) for 6 h [21], Behavioral, electrophysiological, and biochemical tests were performed at 6 h after injection (Fig. 1A). The results from open field experiment showed that there was no significant difference in the total distance between the LPS group and control group (Fig. 1B). The Novel Object Recognition (NOR) test showed that the exploration duration of new object after LPS treatment was significantly reduced at 2 h (Fig. 1C) and 24 h (Fig. 1D). Fear condition results showed that the freezing duration after LPS treatment was significantly lower than the control group at 2 h (Fig. 1E) and 24 h (Fig. 1F). Finally, Morris water maze (MWM) was used to test memory and learning abilities, and the result showed that the LPS rats have significantly increased latency to find the hidden platform (Fig. 1G). On the day 6, the platform was removed to test the spatial memory, both the time in the target quadrant (Fig. 1H) and the number of target platform crossing (Fig. 1I) were decreased. Taken together, these results suggest that LPS causes learning and memory impairments.

### LPS treatment induces synaptic dysfunction in rats

**Fig. 2:**
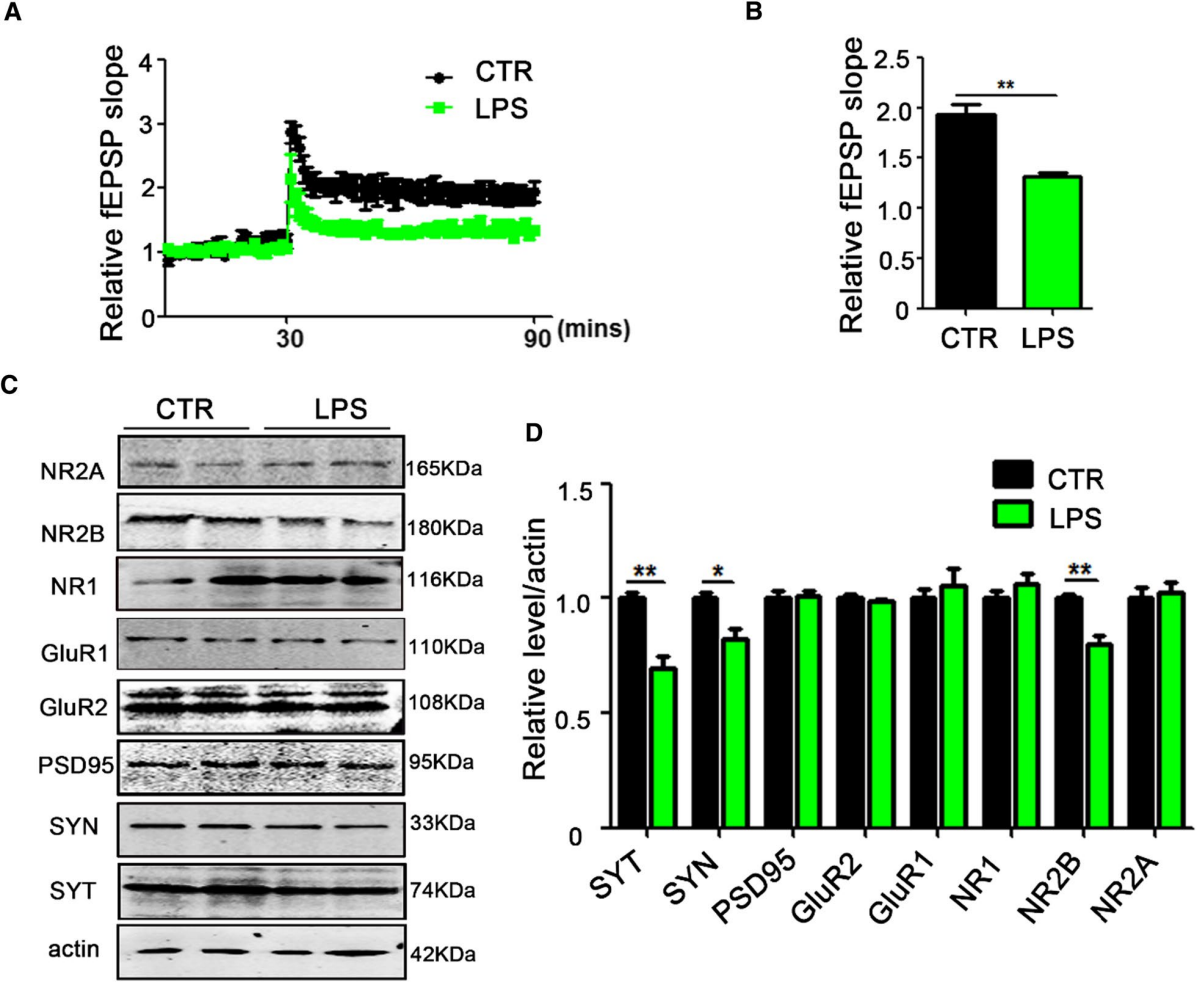
LPS treatment induces synaptic dysfunction in rats. **A**, **B** Hippocampal CA3-CA1 LTP and its quantification (**A**) were recorded using the MED64 system. Normalized CA3-CA1 fEPSP mean slope recorded from the CA1 dendritic region in hippocampal slices (**B**). **C**, **D** Brain tissues from hippocampi were homogenized, and synaptic protein levels were detected by immunoblotting. n = 3. *p* value significance is calculated from a *T*-test, and data are presented as mean ± SEM. *p < 0.05, **p < 0.01 vs control group

A large number of studies have shown that the hippocampus (HIP) plays a very important role in learning and memory function [30–32]. To investigate how LPS induces learning and memory deficits, we explored whether hippocampal-dependent synaptic plasticity was impaired by recording long-term potentiation (LTP). The LTP test showed that the field excitatory postsynaptic potential (fEPSP) slope of the LPS group was decreased after high frequency stimulation (HFS) (Fig. 2A and B). In addition, we examined molecular changes in synapse-related proteins in the hippocampus. Western blotting (Fig. 2C) results showed that the down-regulation of presynaptic proteins synapsin (SYN) and synaptophysin (SYT) and postsynaptic protein NR2B in the LPS group (Fig. 2D). This is an indication that LPS leads to cognitive dysfunction through the alteration of synaptic function seen as decrease of both synaptic proteins and fEPSP.

### LPS treatment results in synaptic dysfunction in rat primary hippocampal neurons

**Fig. 3:**
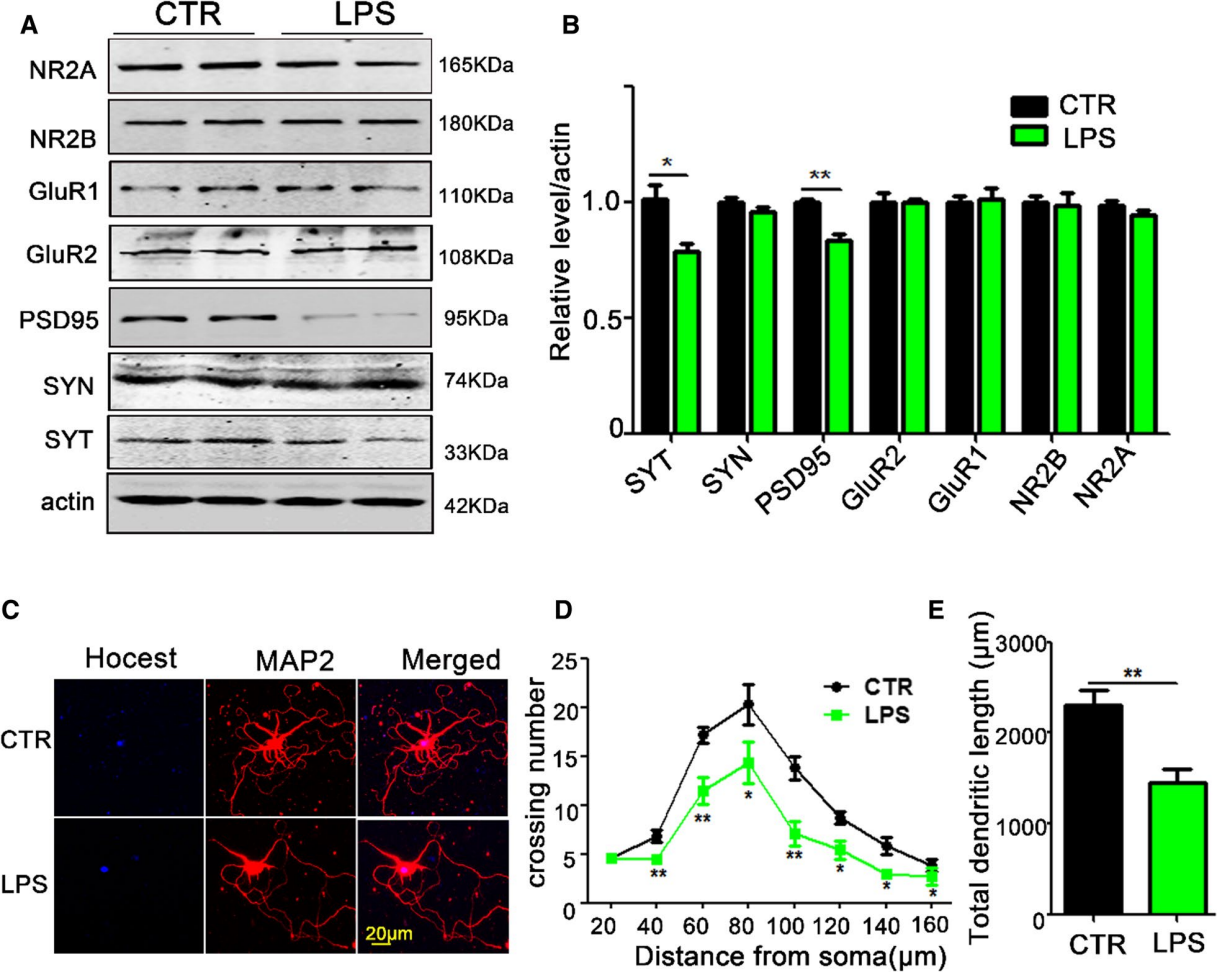
LPS treatment results in synaptic dysfunction in rat primary hippocampal neurons. Western blotting (**A**) from homogenized primary hippocampal neurons showed the levels of synaptic protein detected by immunoblotting (**B**), n = 3. **C**–**E** Rat primary hippocampal neurons (DIV7) were incubated with LPS (1 μg/ml) for 6 h, and the dendritic morphology of hippocampal primary neurons following treatment with LPS was examined using anti-MAP2 antibody (**C**). The dendritic arborization complexity was tested by sholl analysis (**D**), and the total dendritic length was measured (**E**), n = 10. *p* value significance is calculated from a *T*-test, and data are presented as mean ± SEM. *p < 0.05, **p < 0.01 vs control group

LPS treatment induced cognitive impairments in rats, but whether or not it resulted in neuronal damage has not been clarified. To investigate the underlying mechanism based on synaptic morphology, primary hippocampal neurons were treated with LPS (1 μg/ml) for 6 h [33]. Western blotting showed a downregulation in the levels of pre-synaptic proteins SYT and postsynaptic protein PSD95 (Fig. 3A and B). In addition, anti-MAP2 antibody was used to evaluate neuronal morphology (Fig. 3C). When compared with the control group, the complexity of dendritic branches was reduced after LPS treatment at all points above 40 µm away from the cell body (Fig. 3D), as well as the total length of dendrites (Fig. 3E). These results suggest that LPS-induced cognitive impairments could be the reflection of synapticalterations.

### Metformin pretreatment attenuates LPS-induced synaptic dysfunction in rat primary hippocampal neurons

**Fig. 4:**
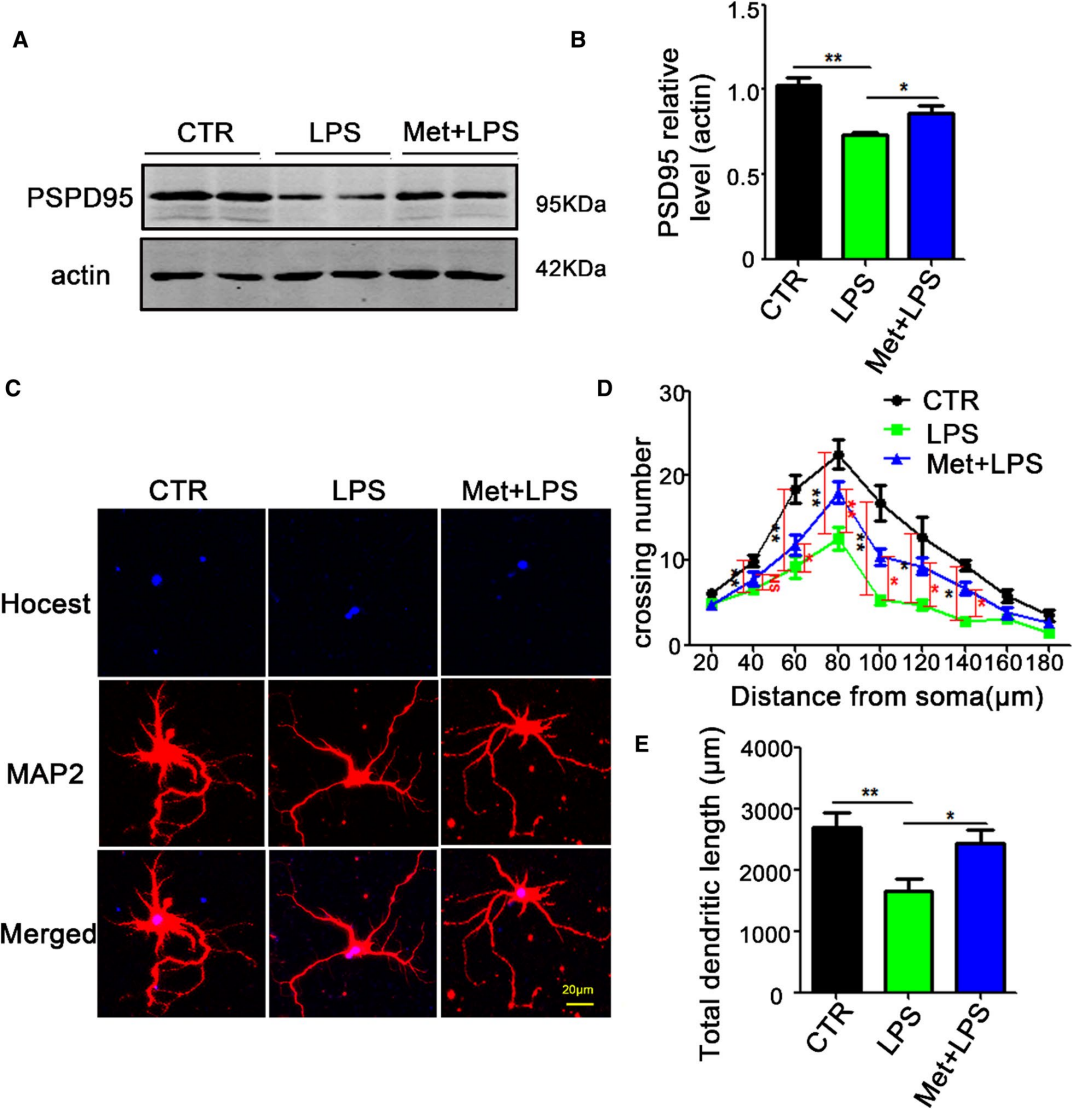
Metformin attenuates LPS-induced synaptic dysfunction in rat primary hippocampal neurons. **A**, **B** A metformin concentration of 5 mM was used to treat primary hippocampal neurons prior to their stimulation with LPS (1 μg/ml) for 6 h. Western blotting was used to test the levels of postsynaptic protein PSD95 (**A**, **B**), n = 3. **C**–**E** Hippocampal primary neurons were pretreated with 5 mM of metformin and treated with LPS, then MPA2 antibody was used to examine dendritic morphology (**C**, **D**), and the total dendritic length (**E**), n = 10. *p* value significance is calculated from a one-way ANOVA or two-way ANOVA tests, all data present mean ± SEM. *p < 0.05, **p < 0.01 vs LPS group

Metformin has shown promise for cognitive impairments in the treatment of diabetes. We therefore, hypothesized that metformin might alleviate LPS-inducedsynaptic dysfunction. To investigate the role of metformin in rat primary hippocampal neurons with LPS treatment, metformin 5 mM was used to pretreat the primary hippocampal neurons prior to their stimulation with LPS (1 μg/ml) for 6 h [33, 34]. Western blotting results showed that metformin attenuated LPS-induced down-regulation of PSD95 (Fig. 4A and B). Immunofluorescence results (Fig. 4C) showed the dendritic complexity at all points farther than 60 µm from the cell body was increased in metformin pretreated neurons prior to LPS when compared with the LPS group (Fig. 4D), as well as an increase in the total dendritic length (Fig. 4E). These data indicate that the metformin rescued the LPS-induced synaptic dysfunction, supporting that metformin might be a potential therapeutic agent for neuroinflammation-induced neurodegenerative diseases.

To investigate the effect of metformin on cognition and the molecular mechanisms in treated and untreated control animals. 5 mM and 10 mM metformin were injected into the lateral ventricles. We firstly performed behavior tests. NOR test results showed a significant increase in the curiosity toward new things in the metformin group when compared with the normal untreated rats (Additional file 1: Fig. S1A, B). Fear condition results showed that the freezing duration after metformin treatment was significantly higher than the untreated control group at 24 h (Additional file 1: Fig. S1D), but there was no significant difference at 2 h (Additional file 1: Fig. S1C). Finally, Morris water maze (MWM) results showed that there were no significantly difference in latency during the 5 training days (Additional file 1: Fig. S1E). On the day 6, with the platform removed to test the spatial memory, both the time in the target quadrant (Additional file 1: Fig. S1F) and the number of target platform crossing were increased (Additional file 1: Fig. S1G) compared with the control group. Moreover, the electrophysiology experiment results showed that metformin enhanced the slope of field excitatory postsynaptic potential (fEPSP) after high-frequency stimulation (HFS) compared with the untreated control group (Additional file 1: Fig. S1H, I). Together, these data suggest that metformin might enhance cognitive function and synaptic plasticity.

### Metformin alleviates LPS-induced cognitive impairments and improves synaptic plasticity

**Fig. 5:**
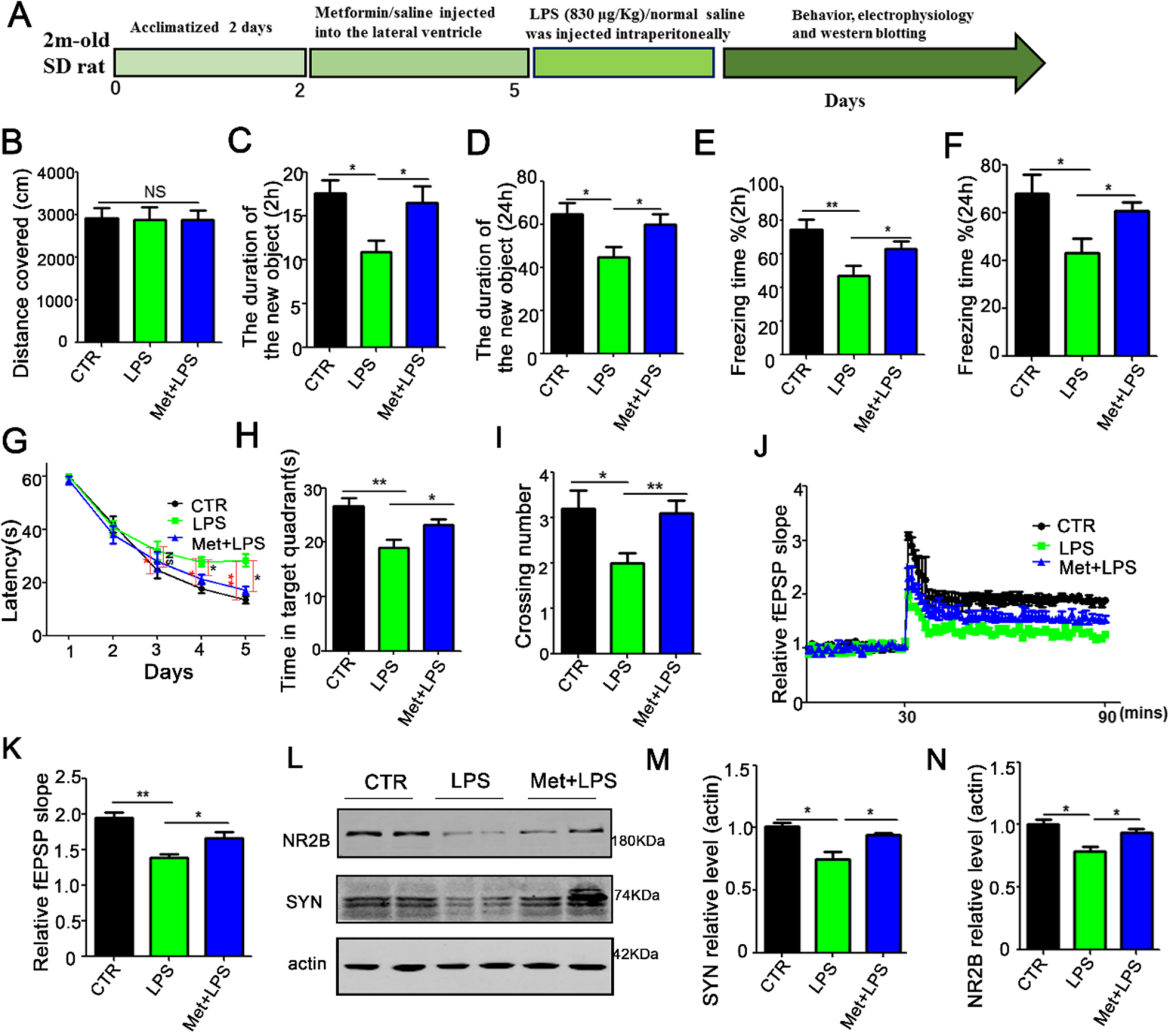
Metformin alleviates LPS-induced cognitive impairments and improves synaptic plasticity. **A** Experimental design sketch. **B** The open field showed no difference in the total distance covered. **C**, **D** Novel object recognition test (NOR) showed the measured recognition index of the new object in 2 h (**B**) and 24 h (**D**). **E**, **F** Fear conditioning test showed the freezing time for 2 h (**E**) and 24 h (**F**). Morris water maze (MWM) result showed that latency to find the hidden platform (**G**). On the day 6, we removed the platform to test the spatial memory, the time in the target quadrant (**H**), and the number of target platform crossing were evaluated (**I**). n = 10. **J**, **K** CA3-CA1 fEPSP mean slope recorded from the CA1 dendritic region in hippocampal slices, n = 3. **K**–**M** Hippocampal tissues were homogenized, and synaptic protein levels were detected by immunoblotting (**L**). Pre-synaptic proteins SYN (**M**) and postsynaptic proteins NR2B (**N**) were analyzed. n = 3. *p* value significance is calculated from a one-way ANOVA or two-way ANOVA tests, all data represent mean ± SEM. *p < 0.05, **p < 0.01vs LPS group

We have shown that metformin rescued the LPS-induced alteration in synaptic protein as well as restoring dendritic complexity in rat primary hippocampal neurons. Moreover, metformin enhances cognitive function and synaptic plasticity in rats. We thus, hypothesized that metformin might alleviate LPS-induced cognitive impairments. To investigate on it, 30 healthy SD rats were randomly divided into three groups. The control group was injected with normal saline intraperitoneally (1 ml /Kg/i.p, n = 10) for 6 h, and the model group (LPS group) was injected with LPS intraperitoneally (830 μg/Kg/i.p, n = 10). In the intervention group (Met + LPS group), 10 mM metformin was injected into the lateral ventricle, and LPS was injected intraperitoneally for 6 h prior to behavioral tests. Behavioral, electrophysiological, and biochemical tests were performed at 6 h after injection (Fig. 5A). The open-field experiment results showed no significant difference in the total distance among all three groups (Fig. 5B). However, the novelty recognition experiment results showed that the Met + LPS group has a significant increase in the duration to explore new things for both 2 h (Fig. 5C) and 24 h (Fig. 5D). Next, fear condition experiment results showed that when compared with the LPS group, freezing duration was significantly improved in the Met + LPS group at 2 h (Fig. 5E) and 24 h (Fig. 5F). Also, Morris water maze (MWM) was used to test memory and learning abilities, and the results showed that the Met + LPS group exhibited significantly decreased latency to find the hidden platform (Fig. 5G). On the day 6, we removed the platform to test the spatial memory, the time in the target quadrant of the Met + LPS group was increased, compared with the LPS group (Fig. 5H), as well as increase in the number of target platform crossing (Fig. 5I). These data suggest that metformin is effective in restoring learning and memory impairments in LPS rats.

We further want to explore whether metformin could improve synaptic plasticity. The LTP test showed that in LPS group, the slope of fEPSP was decreased after high frequency stimulation when compared with the control group. However, the slope of fEPSP was significantly rescued in the Met + LPS group (Fig. 5J, K).In addition, western blotting was used to detect synaptic proteins in the hippocampus (Fig. 5L), and found that the levels of SYN (Fig. 5M) and NR2B (Fig. 5N) in the Met + LPS group were both similar to control group while they were significantly reduced in the LPS group. These results suggest that metformin could reduce LPS-induced synaptic damage and improve cognitive impairments.

### Metformin pretreatment attenuates the release of inflammatory factors via preventing the activation of the NF-κB pathway

LPS treatment upregulates the release of inflammatory factors via activating NF-κB pathway.

Neuroinflammation induces or exacerbates neurodegenerative diseases via activation of microglia cells, which releases various cytokines and other immune factors [9, 33]. To explore whether LPS can induce the release of these various immune factors, we used enzyme-linked immunosorbent assay (ELISA) Kits to detect these factors. The results showed that the LPS could cause a significant increase in the inflammatory cytokines, such as IL-1β (Additional file 1: Fig. S2A), IL-6 (Additional file 1: Fig. S2B), TNF-α (Additional file 1: Fig. S2C), which can trigger acute inflammatory reactions. Interestingly, it was shown that activation of NF-κB mediates the translocation of p65 from the cytoplasm to the nucleus, which in turn mediates immune responses and the release of pro-inflammatory mediators. Therefore, we further explored whether LPS upregulated NF-κB pathway. To this end, we performed western blotting and the results showed that NF-κB p65 subunit has translocated from cytoplasm (Additional file 1: Fig. S2D, E) to nucleus (Additional file 1: Fig. S2F, G) after 6 h of LPS treatment. These results suggest that LPS can activate the NF-κB signaling pathway and then upregulate the release of inflammatory factors.

**Fig. 6:**
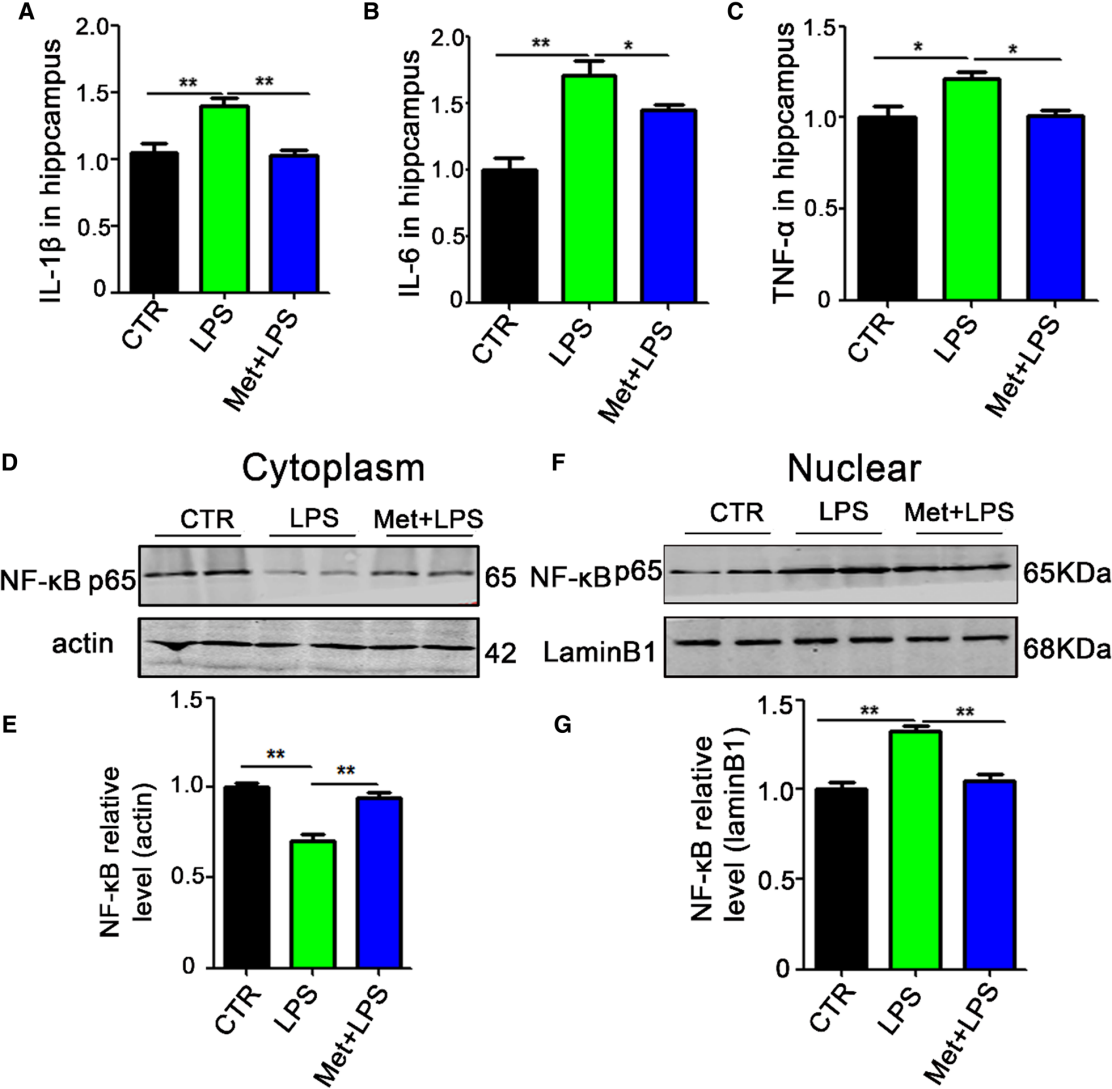
Metformin pretreatment attenuates the release of inflammatory factors via preventing the activation of the NF-κB pathway. **A**–**C** Enzyme-linked immunosorbent assay (ELISA) Kits was used to detect inflammatory factors including IL-1β (**A**), IL-6 (**B**), and TNF-α (**C**). We separate cytosolic and nuclear proteins: NF-κB in the cytoplasm (**D**, **E**) and nucleus (**F**, **G**) was measured. n = 3. *p* value significance is calculated from a one-way ANOVA test, all data represent mean ± SEM. *p < 0.05, **p < 0.01 vs LPS group

We have proposed that LPS leads to nucleus translocation of NF-κB subunit and triggers the expression of various pro-inflammatory mediators. To explore the mechanism of restorative effect of metformin on learning and memory in Met + LPS rats, enzyme-linked immunosorbent assay (ELISA) Kits were used to detect inflammatory factors. The results showed that metformin can significantly inhibit the release of inflammatory factors, such as IL-1β (Fig. 6A), IL-6 (Fig. 6B), and TNF-α (Fig. 6C). We also separate cytosolic and nuclear fractions to explore the effect of metformin on the nucleus translocation of NF-κB by western blotting. The results showed that in the cytosolic fraction from Met + LPS group, the protein level of NF-κB was significantly increased compared to the LPS group (Fig. 6D, E). Furthermore, the nuclear protein level of NF-κB was decreased in Met + LPS group compared to the LPS group (Fig. 6F, G). These data suggest that metformin attenuates LPS-induced neuronal injury and cognitive impairments by blocking NF-κB pathway.

## Discussion

Neuroinflammation is considered to be an important risk factor for the development of neurodegenerative diseases, characterized by cognitive dysfunction, neuronal damage, and apoptosis [35, 36]. It has been reported that neuroinflammation could result in cognitive impairments as a result of nuclear retention of NF-κB p65 and release of inflammatory cytokines. Therefore, in order to explore NF-κB activation and cognitive function, LPS was used in SD rats and primary hippocampal neurons to induce inflammatory response. We found that LPS treatment triggered NF-κB activation. Western blotting results showed that LPS treatment upregulated the translocation of p65 from the cytoplasm to the nucleus.

Metformin obviously decreased LPS-induced nucleus NF-κB p65 translocation. Moreover, the expression of TNF-a, IL-1β, IL-6 were also significantly decreased by metformin. However, it remains to be confirmed whether metformin alleviates synaptic damage and cognitive impairments. To address this, primary hippocampal neurons were incubated with or without metformin prior to LPS treatment. The results showed that LPS significantly increased neuronal injury. While the pretreatment of primary neurons with metformin obviously reduce neuronal injury. In addition, rats that were intraperitoneally injected with LPS following intraventricular injection with metformin showed enhanced cognitive function compared to those with LPS injection alone.

## Conclusion

Conclusively, our data strongly support the notion that metformin attenuates LPS-induced synaptic dysfunction and cognitive impairments in rats by blocking NF-κB pathway. To our knowledge, this is the first study to explore the mechanism underlying the neuron damage caused by NF-κB pathway alterations. The present findings provide an insight that further clarify the mechanism of the neuroinflammation-induced neurotoxicity.

## Supplementary Information

**Additional file 1: Figure S1.** Metformin might be beneficial to cognitive function and synaptic plasticity. **Figure S2.** LPS treatment upregulates the release of inflammatory factors via activating NF-κB pathway.

## Data Availability

The datasets used and/or analyzed during the present study are available from the corresponding author on reasonable request.

## Abbreviations

PD: Parkinson’s disease
MS: Multiple sclerosis
AD: Alzheimer’s disease
LPS: Lipopolysaccharides
SD: Sprague–Dawley
Hip: Hippocampus
Met: Metformin
NO: Nitric oxide
ROS: Reactive oxygen species
LTP: Long-term potentiation
SC: Schaffer collateral
fEPSP: Field excitatory postsynaptic potential
HFS: High-frequency stimulation
PSD95: Postsynaptic density protein 95
SYN: Synapsin
SYT: Synaptophysin
ELISA: Enzyme-linked immunosorbent assay
IL-1β: Interleukin-1β
IL-6: Interleukin-6
TNF-α: Tumor necrosis factor-α
i.p: Intraperitoneally
CA1: Cornu Ammonis area 1
CA3: Cornu Ammonis area 3
MWM: Morris water maze
NOR: Novel objective recognition
