# Supplementary material for: Metformin attenuates LPS-induced neuronal injury and cognitive impairments by blocking NF-κB pathway
Source: BMC Neurosci. 2021 Nov 26;22:73. doi: 10.1186/s12868-021-00678-5 (PMC8626880; doi:10.1186/s12868-021-00678-5)
Supplement: Supplementary file 1 — Additional file 1: Figure S1. Metformin might be beneficial to cognitive function and synaptic plasticity. Figure S2. LPS treatment upregulates the release of inflammatory factors via activating NF-κB pathway. [file 12868_2021_678_MOESM1_ESM.docx]

**Additional file 1**

**Figure S1**

**
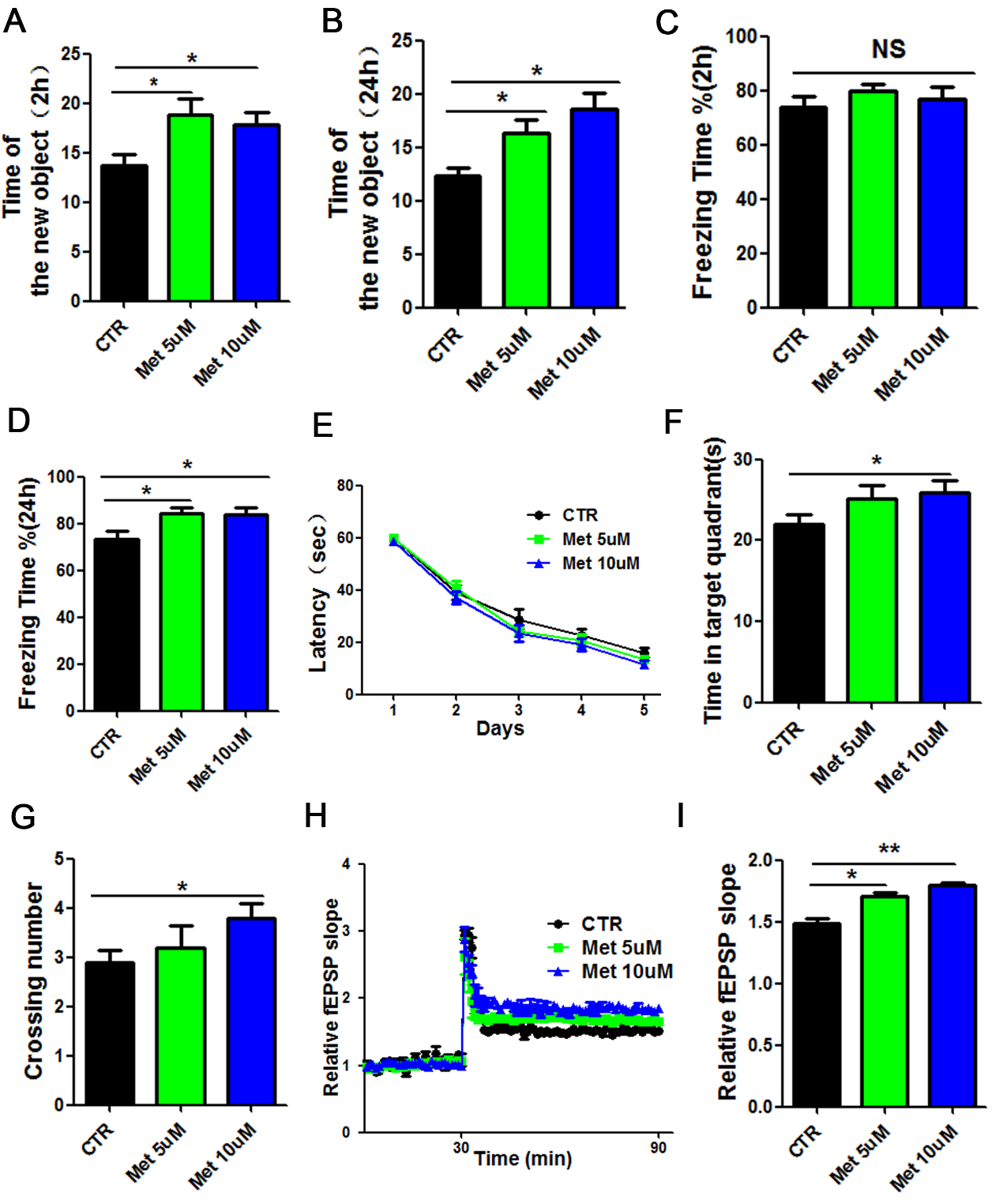
**

**Figure S2**


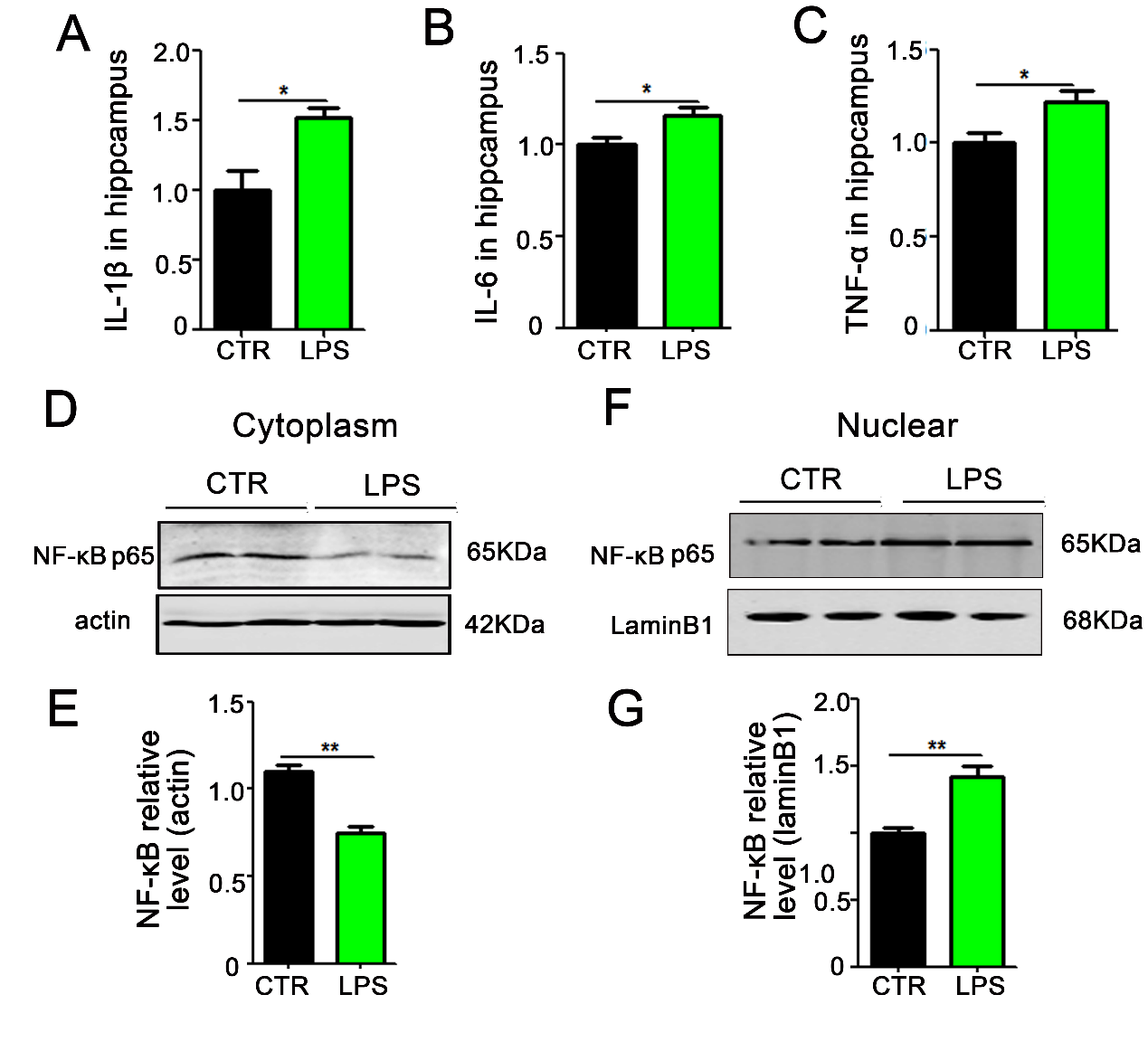


**Figure legend**

**Figure S1. Metformin might be beneficial to cognitive function and synaptic plasticity.**

Metformin concentrations of 5mM and 10mM were injected into the lateral ventricles to explore the role in normal untreated animal. NOR showed the measured recognition index of the new object in 2hours (A) and 24hours (B). (C, D) Fear conditioning test showed the freezing time for 2hours (C) and 24hours (D). Morris water maze (MWM) result showed that latency to find the hidden platform (E). On the day 6, we removed the platform to test the spatial memory, the time in the target quadrant (F), and the number of target platform crossing (G) were measured. n=10. (H, I) CA3-CA1 fEPSP mean slope recorded from the CA1 dendritic region in hippocampal slices, n=3. *p* value significance is calculated from a one-way ANOVA test, and data are presented as mean ± SEM. **p* < 0.05, ***p* < 0.01 *vs* control group.

**Figure S2.** **LPS treatment upregulates the release of inflammatory factors via activating NF-κB pathway.**

(A-C) Enzyme-linked immunosorbent assay (ELISA) Kits were used to detect inflammatory factors including IL-1β (A), IL-6 (B), TNF-α (C). We separate cytosolic and nuclear proteins: NF-κB in the cytoplasm (D, E) and nucleus (F, G) was measured. n=3. *p* value significance is calculated from a T-test, and data are represented as mean ± SEM. **p* < 0.05, ***p* < 0.01 *vs* control group.
